# Myeloperoxidase Inhibition Decreases the Expression of Collagen and Metallopeptidase in Mare Endometria under In Vitro Conditions

**DOI:** 10.3390/ani11010208

**Published:** 2021-01-16

**Authors:** Ana Amaral, Carina Fernandes, Maria Rosa Rebordão, Anna Szóstek-Mioduchowska, Karolina Lukasik, Pedro Pinto-Bravo, Luís Telo da Gama, Dariusz Jan Skarzynski, Graça Ferreira-Dias

**Affiliations:** 1 CIISA—Centro de Investigação Interdisciplinar em Sanidade Animal, Departamento de Morfologia e Função, Faculdade de Medicina Veterinária, Universidade de Lisboa, 1300-477 Lisboa, Portugal; nita.amaral@gmail.com (A.A.); fachica@hotmail.com (C.F.); milorebordao@gmail.com (M.R.R.); ltgama@fmv.ulisboa.pt (L.T.d.G.); 2 Polytechnic of Coimbra, Coimbra Agriculture School, Bencanta, 3045-601 Coimbra, Portugal; pbravo@esac.pt; 3 Institute of Animal Reproduction and Food Research, Polish Academy of Science, 10-748 Olsztyn, Poland; a.szostek-mioduchowska@pan.olsztyn.pl (A.S.-M.); k.lukasik@pan.olsztyn.pl (K.L.); d.skarzynski@pan.olsztyn.pl (D.J.S.)

**Keywords:** endometrosis, myeloperoxidase, 4-aminobenzoic acid hydrazide, fibrosis, metallopeptidases

## Abstract

**Simple Summary:**

Myeloperoxidase, which is released by neutrophils when they form neutrophil extracellular traps, has already been related to equine endometrosis development. The myeloperoxidase inhibitor 4-aminobenzoic acid hydrazide (ABAH) has shown promising results inhibiting myeloperoxidase in other pathological conditions. The metallopeptidases (MMP-2/-9) regulates the extracellular matrix turnover. In the present study, in equine explants from follicular phase, myeloperoxidase treatment increased collagen type I relative protein abundance, but the use of ABAH reduced it. In mid-luteal phase endometrial explants, MMP-2 seems to be implicated in an acute reaction to myeloperoxidase treatment, while in the follicular phase, MMP-9 might be associated with a prolonged exposition to myeloperoxidase. The impairment of equine endometrosis can be achieved by inhibiting the pro-fibrotic effects of myeloperoxidase using the inhibitor ABAH.

**Abstract:**

Neutrophils can originate neutrophil extracellular traps (NETs). Myeloperoxidase (MPO) is a peroxidase found in NETs associated to equine endometrosis and can be inhibited by 4-aminobenzoic acid hydrazide (ABAH). Metallopeptidases (MMPs) participate in extracellular matrix stability and fibrosis development. The objectives of this in vitro work were to investigate, in explants of mare’s endometrium, (i) the ABAH capacity to inhibit MPO-induced collagen type I (COL1) expression; and (ii) the action of MPO and ABAH on the expression and gelatinolytic activity of MMP-2/-9. Explants retrieved from the endometrium of mares in follicular or mid-luteal phases were treated with MPO, ABAH, or their combination, for 24 or 48 h. The qPCR analysis measured the transcription of *COL1A2*, *MMP2*, and *MMP9*. Western blot and zymography were performed to evaluate COL1 protein relative abundance and gelatinolytic activity of MMP-2/-9, respectively. Myeloperoxidase elevated COL1 relative protein abundance at both treatment times in follicular phase (*p* < 0.05). The capacity of ABAH to inhibit MPO-induced COL1 was detected in follicular phase at 48 h (*p* < 0.05). The gelatinolytic activity of activated MMP-2 augmented in mid-luteal phase at 24 h after MPO treatment, but it was reduced with MPO+ABAH treatment. The activity of MMP-9 active form augmented in MPO-treated explants. However, this effect was inhibited by ABAH in the follicular phase at 48 h (*p* < 0.05). By inhibiting the pro-fibrotic effects of MPO, it might be possible to reduce the development of endometrosis. Metallopeptidase-2 might be involved in an acute response to MPO in the mid-luteal phase, while MMP-9 might be implicated in a prolonged exposition to MPO in the follicular phase.

## 1. Introduction

Myeloperoxidase (MPO) is an enzyme that is expressed by several immune cells, such as neutrophils, monocytes, and macrophages [1,2]. Neutrophils are the first leucocytes acting on the defense against microbial attacks [3,4] and are able to degranulate and release their DNA and some enzymes that possess antimicrobial properties. Thus, they form neutrophil extracellular traps (NETs). Some proteases, such as cathepsin G and elastase, and the peroxidase MPO are released by NETs to fight bacteria [5]. Among these enzymes, MPO has been described to be the most abundant one in neutrophils [6]. It uses the bacteria-induced hydrogen peroxide to produce chloramine and hypochlorite, which are toxic products for bacteria [3,7].

After mating or artificial insemination, the sperm induces inflammation with a rapid influx of neutrophils into the uterus, which in turn leads to a physiological transient breeding-induced endometritis [8,9]. This inflammatory response results in the elimination of needless spermatozoa, contaminating bacteria, and debris introduced in the uterus [10,11]. The process of NETs formation in equine endometrium has already been demonstrated by the contact of equine neutrophils with semen [12,13] or with bacteria associated with endometritis [14]. However, besides the antimicrobial properties of NETs components, they may also contribute to the development of some pathological conditions [15]. High concentrations of MPO in uterine lavage of mares was already related with endometritis [16], even though this was not demonstrated in cows with endometritis [17]. In our previous studies, we have also identified the involvement of NETs proteases in the establishment of endometrosis [18,19,20,21]. Endometrosis is a fibrotic, progressive, and degenerative condition, mainly diagnosed on the grounds of the paramount pathologic deposition of extracellular matrix (ECM) proteins, such as collagen, in mare endometrium [22,23]. In fact, the treatment of equine endometrial explants with NETs proteases induced collagen type I (COL1) expression [18,19,20,21]. In humans, elevated levels of MPO in cystic fibrosis sputum have been associated with the severity of lung disease [24,25]. Moreover, this enzyme has also been linked to liver fibrosis [26,27].

The 4-aminobenzoic acid hydrazide (ABAH), among many MPO inhibitors tested, has been the most investigated [28,29]. Recent studies have shown that ABAH reduced MPO-dependent mice hepatocyte death [27], decreased the activity of MPO in acute stroke in mice [30], and inhibited MPO in cystic fibrosis sputum in humans [31].

Metallopeptidases (MMPs) are a group of enzymes that mediate ECM turnover. Metallopeptidase-2 and -9 are endopeptidases that denature ECM substrates, such as collagens (gelatins) [32]. In addition, the expression and activity of MMPs are affected by ovarian hormones in human endometrial tissue remodeling during the estrous cycle phases [33], but their role in fibrosis establishment is still uncertain. Comparing the normal and fibrotic equine endometrium, Aresu et al. [34] found no alteration in MMP-2/-9 expression. However, MMP-2 transcription [35] and its active form [36] were up-regulated in mare endometrosis. Our latest in vitro studies in equine endometrium revealed that the expression of MMPs is altered by factors involved in inflammation [transforming growth factor (TGF) β1, interleukins and prostaglandins] [37,38,39], and could contribute to the fibrotic response to elastase [20] and cathepsin G [21].

Taking into consideration that equine endometrial explants treated with enzymes found in NETs induced COL1, it could be proposed that NETs enzymes play a role in equine endometrial fibrosis establishment. Thus, the rationale was to investigate if inhibiting NETs enzymes would reduce the MPO induced COL1. This way, we proposed to evaluate if a selective inhibitor of NETs enzyme MPO would be effective in reducing MPO pro-fibrotic effect, as it has been shown in other organs and species. Our previous in vitro findings in mare endometrial explants showed that COL1 induced by elastase and cathepsin G was reduced by the use of their selective inhibitors [20,21]. We have hypothesized that by inhibiting MPO, the in vitro production of COL1 by mare endometrial explants would be reduced. Thus, the objectives of this in vitro work were to investigate, in explants of mares’ endometrium, (i) the ABAH capacity to inhibit MPO-induced collagen type I (COL1) expression; and (ii) the action of MPO and ABAH on the expression and gelatinolytic activity of MMP-2/-9.

## 2. Materials and Methods

### 2.1. Mares and Retrieval of Endometrium

Uteri from cyclic mares (*n* = 14) intended for meat production were collected *post-mortem* at an abattoir (Rawicz, Poland) within 10–15 min of the mares’ euthanasia, in agreement the European (EFSA, AHAW/04–027) legislation. As confirmed by the inspection carried out by the official veterinary doctor, the mares used in this study showed no signs of illness. For further progesterone (P4) analysis, blood from the jugular vein was withdrawn into ethylenediaminetetraacetic acid (EDTA) tubes. For each mare, the estrous cycle phase was determined according to uterine and ovarian evaluation and confirmed by P4 concentration in plasma [18,40]. For this study, mid-luteal phase (MLP; *n* = 6) and follicular phase (FP; *n* = 8) endometria were immediately transported on ice to the laboratory. As previously reported, mare uteri were placed in ice-cold Dulbecco’s modified Eagle’s medium (DMEM) F-12 Ham medium (D/F medium; 1:1 (*v*/*v*); D-2960; Sigma-Aldrich, St Louis, MO, USA), supplemented with 100 µg/mL streptomycin (S9137; Sigma-Aldrich), 100 IU/mL penicillin (P3032; Sigma-Aldrich) and 2 µg/mL amphotericin (A2942; Sigma-Aldrich). Only endometria without endometritis were included in the study, as previously referred [18,41]. After samples were collected, two pieces of endometrium were immersed in 4% buffered paraformaldehyde for the histopathological classification of the endometrium [42]. The endometrial samples were classified as I, IIA, IIB, or III categories based on the extent of inflammation and/or fibrosis, according to Kenney and Doig [42]. Only mares presenting mild to moderate histopathological lesions (IIA or IIB category) were considered in this experiment to exclude the variation that is due to endometrial fibrotic grade.

### 2.2. In Vitro Culture of Endometrial Explants

Endometrial explants preparation and culture were performed as reported before [20]. The explants were pre-incubated for 1h at 38 °C, 5% CO_2_, in a chamber with a humidified atmosphere (Biosafe Eco-Integra Biosciences, Chur, Switzerland) in 24-well cell culture sterile plates (Eppendorf, #0030 722.116) with 1mL of DMEM culture medium supplemented with 0.1% (*w*/*v*) bovine serum albumin (BSA; 735078; Roche Diagnostics, Mannheim, Germany), 2 µg/mL amphotericin (A2942; Sigma-Aldrich), 100 IU/mL penicillin (P3032; Sigma-Aldrich), and 100 µg/mL streptomycin (S9137; Sigma-Aldrich) with gentle shaking (150 rpm). Afterwards, endometrial explants (FP: *n* = 8; MLP: *n* = 6 for all the performed treatments) were further treated in new culture medium for 24 h or 48 h with (i) vehicle (negative control)–culture medium alone; (ii) myeloperoxidase (MPO; 0.5 µg/mL; orb81997; Biorbyt, Cambridge, UK); (iii) 4-aminobenzoic hydrazide, an MPO inhibitor (ABAH; 10 µg/mL; C7H9N3O, sc-204107; Santa Cruz Biotechnology, Dallas, TX, USA); (iv) MPO (0.5 µg/mL) + ABAH (10 µg/mL); or (v) oxytocin (OXT; 10-7 M). Oxytocin was previously used to induce prostaglandin (PG) secretion in equine endometrial explants [43,44]. Thus, in the present study, OXT treatment was a means to determine explant viability by assessing endometrium in vitro capacity to secrete PGF2α throughout the incubation time. A fibrogenic assay using TGFβ1 was previously carried out as a positive control for COL expression [20]. Each treatment was carried out in quadruplicate. When the culture media were replaced, 1 h after pre-incubation, ABAH was added. One hour later, MPO was added to allow binding of the inhibitor. In studies lasting for 48 h, 10 µg/mL of ABAH were furthered added after the first 24 h of treatment since in the pre-trial its inhibitory effect persisted only for 24 h and subsided afterwards. In the end, explants (in RNAlater, R901, Sigma-Aldrich) and conditioned culture media were stored at −80 °C. For PG analysis, a 1% stabilizer solution of 0.3M EDTA (E5134, Sigma-Aldrich) and 1% aspirin (A2093; Sigma-Aldrich) was added to the culture medium to prevent degradation before storage at −80 °C.

The expression of TGFβ1, as a fibrotic indicator [18], was induced by 0.5 µg/mL of MPO in a dose assessment assay. In addition, a dose-response trial based in other in vitro studies [28,45], using 0.01, 0.1, 1, 10 and 100 µg/mL of ABAH, showed that the optimum concentration that inhibited *COL1A2* mRNA MPO-induced was 10 µg/mL (data not shown).

### 2.3. Endometrial Explant Viability Assay

The endometrial explant viability was evaluated by lactate dehydrogenase (LDH) activity and by PGF2α secretion in conditioned culture medium, as described [20]. The data of viability of endometrial explants are presented in Supplementary Material, Figures S1 and S2.

### 2.4. Total RNA Extraction, Synthesis of cDNA and qPCR

TRI Reagent^®^ (T9424; Sigma-Aldrich) was used to perform the extraction of total RNA, following the guidelines provided by the manufacturer. The evaluation of both RNA quality and quantity, as well as cDNA synthesis, was done as already reported [20]. The primer sequences for *COL1A2, MMP2, MMP9,* and for *ribosomal protein L32* (*RPL32*) reference gene were earlier determined (Table 1), as well as the reference gene validation [20,46]. The reference genes tested were *glyceraldehyde 3-phosphate dehydrogenase* (*GAPDH*), *succinate dehydrogenase A complex, subunit A, flavoprotein* (*SDHA*), *beta-2-microglobulin* (*B2M*), and *RPL32*. The *RPL32* reference gene was revealed to be the most stable among the genes tested, showing less than two-fold changes between different biological conditions [46]. The target and reference gene reactions were run simultaneously, in duplicate, on a 96 well plate (4306737; Applied Biosystems) and run in a StepOnePlus™ Real-Time PCR System (Applied Biosystems, Warrington, UK). The specificity of the qPCR products was performed as described [20,47].

### 2.5. Western Blot Analysis

Equine endometrial explants were processed as described before [20]. Collagen type I protein relative abundance was determined by the non-staining total protein loading control method, as reported before [20]. The primary against COL1 antibody (1:1000 diluted; 20121; Novotec, Lyon, France) was incubated overnight at 4 °C, as previously described, and validated [18]. The secondary antibody utilized was Horseradish peroxidase (HRP)-conjugated anti-rabbit (1:20,000; P0448; DakoCytomation, Carpinteria, CA, USA) incubated for 1.5 h at room temperature. Imaging of relative protein abundance of COL1 was achieved by luminol-enhanced chemiluminescence (Super Signal West Pico, 34077; Thermo Scientific, Waltham, MA, USA). For band normalization and comparison of all membranes, a standard sample (30 µg) of a mix of explants was loaded in all gels. Image Lab 6.0 (Bio-Rad, Hercules, CA, USA) software and a multichannel protocol were used to analyze COL1 relative abundance in lanes in non-staining total protein membrane image. After antibodies incubation, the band was detected on chemiluminescence image. The target protein volume was software-calculated using a normalization factor allowing the adjustment of variability of the protein loaded [20,48].

### 2.6. Zymography

The MMP-2 and MMP-9 gelatinolytic activity was assessed by zymography, as described before [49]. Normalization of zymograms was accomplished using a non-staining total protein loading control [50]. The explant culture supernatant was processed, as described [20]. The molecular weight determination was made using Recombinant Human MMP-2 Protein, CF (902-MP-010; R&D Systems, Minneapolis, MN, USA), and Recombinant Human MMP-9 Western Blot Standard Protein (WBC018; R&D Systems). To relate all the gels, a single lane of 40 µg standard sample of a mix of culture media was loaded. A multichannel protocol (Image Lab 6.0, Bio-Rad) was created for the detection of lanes in non-staining total protein gel image and bands on Coomassie staining image. Volume of target protein as well as the normalization factor were calculated and the values adjusted for protein load variation [20,50].

### 2.7. Statistical Analysis

Data normality was evaluated visually and by the Kolmogorov–Smirnov test in Proc Univariate of SAS v. 9.4 (SAS Institute Inc., Cary, NC, USA). The viability data were assessed by one-way analysis of variance (ANOVA) followed by Tukey’s multiple comparisons test (GraphPAD PRISM, Version 6.00, 253 GraphPad Software, San Diego, CA, USA). These results are displayed as mean ± SEM, and significance was determined at *p* < 0.05. The evaluated variables consisted of *COL1A2*, *MMP2* and *MMP9* transcription; relative COL1 protein abundance; and gelatinolytic activity of MMP-2/-9. Since some variables did not present a normal distribution, the square root and logarithmic transformation were used to transform these data for further analysis. In the first analysis, the PROC GLM of SAS was used to analyze each response variable to different treatments, including combination of the effect of MPO, the use of ABAH, estrous cycle phase, and time of treatment, resulting in 16 treatment combinations in total. The least square means of the treatment combinations were compared (using the PDIFF of PROC GLM), and results were significant at *p* < 0.05. To perform the graphical presentation, the means were back transformed. After, the two-, three-, and four-way interactions were also analyzed.

## 3. Results

### 3.1. The Effect of ABAH on the Inhibition of COL1 Induced by MPO

**Figure 1:**
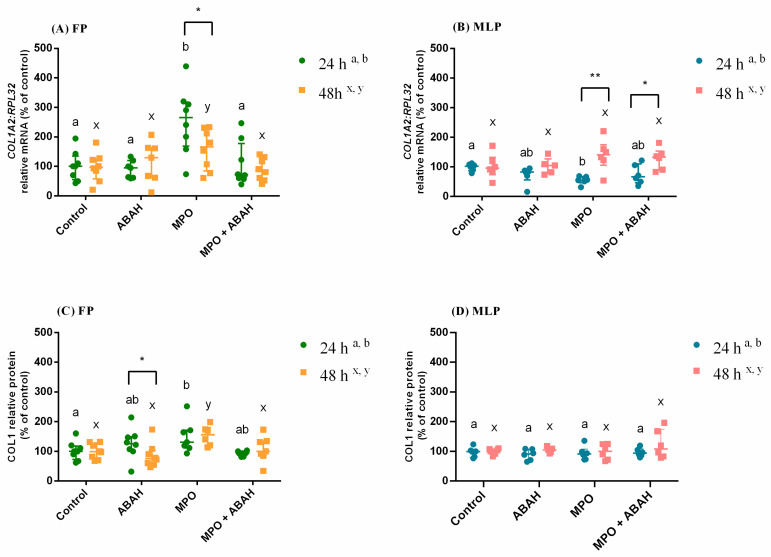
Relative mRNA transcription of type I collagen (*COL1A2*) (**A**,**B**) and relative abundance of COL1 protein (**C**,**D**) in mare endometrial explants from the follicular phase (FP) and the mid-luteal phase (MLP) treated with culture medium only (Control), myeloperoxidase (MPO: 0.5 μg/mL), 4-aminobenzoic hydrazide (ABAH: 10 μg/mL), or MPO (0.5 μg/mL) + ABAH (10 μg/mL) for 24 or 48 h. Results are presented as median with interquartile range. Significance was determined at *p* < 0.05. The differences among treatments with the same treatment time are signaled by distinct superscript letters (a,b—24 h; x,y—48 h). The differences among times of treatment for the identical treatment are shown by asterisks (* *p* < 0.05; ** *p* < 0.01).

**Figure 2:**
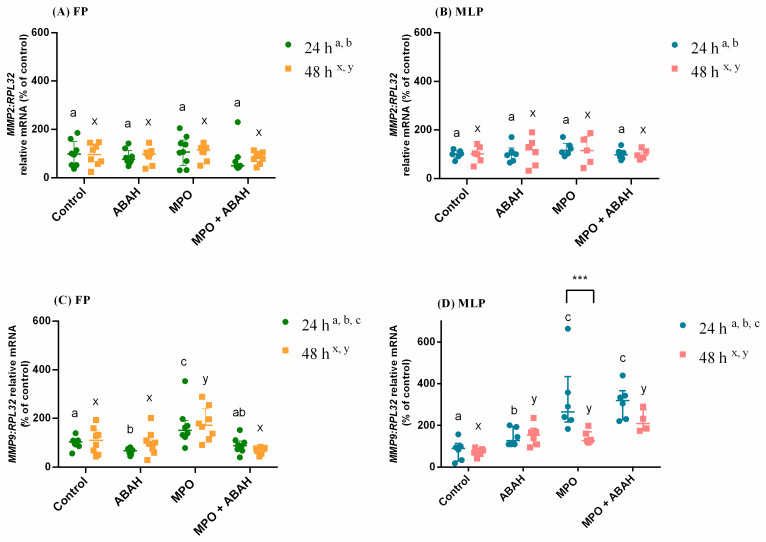
Transcription of *MMP2* (**A**,**B**) and *MMP9* (**C**,**D**) relative mRNA in mare endometrial explants from the follicular phase (FP) and the mid-luteal phase (MLP) treated with culture medium only (Control), myeloperoxidase (MPO: 0.5 μg/mL), 4-aminobenzoic hydrazide (ABAH: 10 μg/mL), or MPO (0.5 μg/mL) + ABAH (10 μg/mL) for 24 or 48 h. Results are presented as median with interquartile range. Significance was determined at *p* < 0.05. The differences among treatments with the same treatment time are signaled by distinct superscript letters (a,b,c—24 h; x,y—48 h). The differences among times of treatment for the identical treatment are depicted by asterisks (*** *p* < 0.001).

**Figure 3:**
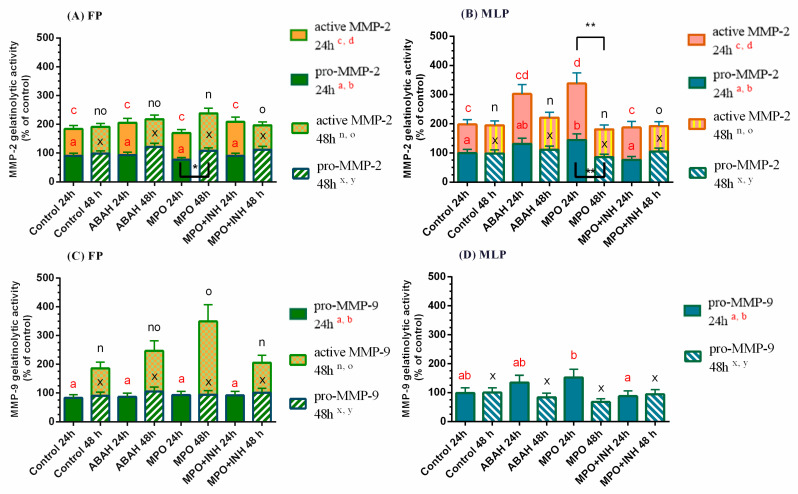
Relative MMP-2 (**A**,**B**) and MMP-9 (**C**,**D**) gelatinolytic activities in mare endometrial explants from the follicular phase (FP) and the mid-luteal phase (MLP) treated with culture medium only (Control), myeloperoxidase (MPO: 0.5 μg/mL), 4-aminobenzoic hydrazide (ABAH: 10 μg/mL), or MPO (0.5 μg/mL) + ABAH (10 μg/mL) for 24 or 48 h. Data of the least square means ± SEM are shown in bars as percentage of change from control. Significance was determined at *p* < 0.05. The differences among treatments with the same treatment time are signaled by distinct superscript letters. The differences among times of treatment for the identical treatment and MMP form are presented by asterisks (* *p* < 0.05; ** *p* < 0.01).

In Figure 1, the relative mRNA transcription and protein abundance of COL1 results are presented as median with interquartile range. Likewise, in Figure 2, the transcription of *MMP2/9* is shown as median with interquartile range. However, in Figure 3, the results of MMP-2/-9 gelatinolytic activities are presented as least square means ± SEM. These figures were drawn in GraphPAD PRISM.

The *COL1A2* transcripts increased in MPO treated explants in FP at 24 h and 48 h when related to the control group (*p* < 0.0001; *p* < 0.05 respectively; Figure 1A), and to the ABAH-treated group (*p* < 0.0001; *p* < 0.05 respectively; Figure 1A). However, the use of MPO and ABAH treatments, when combined, impaired *COL1A2* mRNA levels compared to the corresponding MPO-treated tissues (FP: 24 h—*p* < 0.001; 48h—*p* < 0.01; Figure 1A). In MLP endometrial explants treated for 24 h, the MPO treatment lowered the transcription regarding the control group (*p* < 0.05; Figure 1B). In FP endometrial explants treated with MPO, *COL1A2* transcription was higher at 24 h than at 48 h (Figure 1A). Nevertheless, in MLP explants the transcription was higher at 48 h compared to 24 h (Figure 1B) and also increased with MPO + ABAH treatment (Figure 1B).

In MPO-treated explants, COL1 protein increased in the FP, both at 24 h (*p* < 0.01; Figure 1C) and 48 h (*p* < 0.001; Figure 1C); comparing to the control and ABAH groups, this only occurred during the longest treatment time (*p* < 0.0001; Figure 1C). The inhibitory effect of ABAH was detected in FP at 48 h regarding the group treated with MPO (*p* < 0.01; Figure 1C, Supplementary Material Figure S3). In the FP, COL1 protein relative abundance was greater in ABAH-treated explants at 24 h when compared to 48 h treatment (Figure 1C; Supplementary Material Figure S3). There were no differences in COL1 protein between treatments or treatment times in the MLP explants (Figure 1D).

### 3.2. The Influence of MPO and ABAH on MMP Expression

The transcription of *MMP2* was unchanged at both treatment times and estrous cycle phase (Figure 2A,B). In FP endometrial explants, at both treatment times, the transcript levels of *MMP9* increased in MPO treated groups relative to the corresponding non-treated explants (*p* < 0.01; Figure 2C) and to the ABAH-treated set (24 h—*p* < 0.0001; 48 h—*p* < 0.01; Figure 2C). However, the MPO + ABAH combined treatment reduced *MMP9* mRNA compared to MPO-treated groups (FP: 24 h—*p* < 0.01; 48 h—*p* < 0.0001; Figure 2C). The ABAH treatment decreased MMP-9 mRNA comparing to the non-treated group at 24 h in FP endometria (*p* < 0.05; Figure 2C). In MLP tissues treated for 24 h, all the treatments up-regulated *MMP9* transcription relative to the control (ABAH—*p* < 0.05; MPO and MPO + ABAH—*p* < 0.001; Figure 2D). Moreover, the MPO and MPO + ABAH treatments increased *MMP9* mRNA compared to the ABAH-treated group (*p* < 0.001; Figure 2D). At 48 h, in MLP equine explants, all the treatments increased *MMP9* transcript levels as well, compared to control tissues (ABAH—*p* < 0.01; MPO—*p* < 0.05; MPO + ABAH—*p* < 0.0001; Figure 2D). The *MMP9* transcription was higher in MLP tissues MPO-treated at 24 h than at 48 h (Figure 2D).

In FP explants at 48 h, the MPO + ABAH treatment reduced gelatinolytic activity of activated MMP-2 in comparison to MPO-treated explants (*p* < 0.05; Figure 3A; Supplementary Material Figure S3), which did not increase with respect to the control. The gelatinolytic activity of pro- (*p* < 0.05; Figure 3B) and active (*p* < 0.01; Figure 3B) MMP-2 increased in MLP explants at 24 h with MPO treatment compared to control. However, the combined treatment of MPO + ABAH reduced it in comparison to the MPO-treated group (pro-MMP-2: *p* < 0.01; active MMP-2: *p* < 0.05; Figure 3B; Supplementary Material Figure S3).

The pro-MMP-2 gelatinolytic activity increased at 48 h in FP with MPO treatment with respect to 24 h (Figure 3A), while in MLP both pro- and active MMP-2 gelatinolytic activities increased at 24 h relative to 48 h (Figure 3B; Supplementary Material Figure S3).

The MMP-9 active form gelatinolytic activity was only identified in FP at 48 h treatment, and MPO treatment up-regulated it compared to the control group (*p* < 0.01; Figure 3C). Nevertheless, MPO + ABAH combination reduced the activity of active MMP-9, with respect to MPO-treated explants (*p* < 0.01; Figure 3C; Supplementary Material Figure S3). The analysis of pro-MMP-9 gelatinolytic activity showed its decrease after MPO + ABAH of MLP explant treatment for 24 h compared to MPO-treated explants (*p* < 0.05; Figure 3D), which did not differ from its respective control.

The interactions found between treatments, estrous cycle phase, and time of treatment are listed in Supplementary Table S1. In Supplementary Table S2, the differences of the same treatments between the FP and MLP inside each treatment time are presented.

## 4. Discussion

Since the discovery that neutrophils release NETs secondary to the contact with bacteria [14] and semen [12,13] in mare endometrium and that the enzymes found in NETs act as pro-fibrotic factors in mare endometrosis [18], we aimed to investigate if it would be feasible to reduce in vitro COL production induced by these enzymes by specifically inhibiting them [19,20,21]. In our study, MPO treatment elevated the transcripts of *COL1A2* mRNA and COL1 relative protein abundance at both times of incubation only in FP equine endometria. It is in the FP, under the influence of estrogens, that the mare endometrium is more exposed to the invading bacteria, since the cervix is relaxed and open [51]. Nevertheless, it appears that mares with healthy endometria are capable of defeating bacteria during this phase of the estrous cycle. As such, after induced endometritis with *Escherichia coli* infusion, mares presented lower positive bacteriological cultures and neutrophil counts in endometrial swabs when infused at estrus, rather than at diestrus [52]. However, it is worth noting that it is in the FP that *Streptococcus equi* subspecies *zooepidemicus* (bacteria which cause endometritis in the mare) attach the most to mare endometria with endometrosis [53]. Nevertheless, in spite of the mechanisms for defeating bacteria during estrus, a study on gene expression in biopsies from healthy equine endometrium at different times of the estrous cycle showed that, in the FP, the transcription of *COL1A1* increases until the ovulation day [54]. In contrast, in the luteal phase, gene expression of *COL1A1* is lowered [54]. Similarly, in women, stromal endometrial cells treated in vitro with estrogen showed an increase in the deposition of collagen, while progesterone stimulation resulted in an increase in the breakdown of collagen [55]. These studies might explain why the MPO induced collagen response is only observed in the FP. These findings agree with Rebordão et al.’s work [18], where MPO elevated COL1 production in FP type I/IIA endometria. Some studies in other tissues also linked MPO to tissue damage. Human cystic fibrosis [56] and atrial fibrosis [57], as well as liver fibrosis in a mice model [27], have been associated with MPO-induced tissue injury. In addition, stellate cells from liver were activated by MPO, leading to an in vitro up-regulation of COL1 via the fibrogenic factor TGFβ1 [27]. In equine endometrial explants [18,20] and fibroblasts [58], TGFβ1 was linked to endometrosis by increasing COL1 production. We postulated that MPO may also act via TGFβ1 in the equine endometrium.

The inhibitory action of ABAH on MPO-induced COL1 was detected in FP on mRNA expression at both times of treatment, and on protein relative abundance at the longest treatment time. We have shown for the first time to date that by inhibiting MPO in vitro, COL1 protein decreased in mare endometrium. Nonetheless, the ABAH mechanism of action is not yet well known. Some authors proposed a mechanism of action where ABAH is oxidized by MPO to a radical that reduces MPO to its ferrous intermediate by destroying the MPO heme group. Ferrous MPO reacts with hydrogen peroxide, thereby originating an irreversible inactivation [28,59]. However, data from in vitro studies cannot be directly extrapolated to in vivo treatments [60]. Nevertheless, in vitro systems are a faster approach to predict fibrogenic potential by monitoring the response to pro-fibrotic modulators [61]. Thus, the usage of ABAH as a prophylactic and/or therapeutic means for equine endometrial fibrosis needs further evaluation for its in vivo use.

The proteolysis of the ECM appears to be a crucial occurrence in the inflammatory process and, therefore, on the fibrotic process as well. Increased synthesis/deposition and decreased degradation of ECM components lead to fibrosis [62]. The MMPs are enzymes involved in this ECM turnover [31]. In equine explants, MMP-2 and MMP-9 secretion differed when challenged with cytokines and is dependent on the severity of endometrosis, which may link them to modifications in the endometrium that predispose to fibrosis development [37]. It was demonstrated that TGFβ1 treatment increased MMP-9 secretion in mare endometrial fibroblasts and epithelial cells and that the endometrial MMP expression changes at different categories of endometrosis [38]. In other tissues, the concomitant increased levels of MPO and MMP-2/-9 were also reported in rat temporomandibular joint inflammation [63], in inflamed human dental pulp tissue [64], and in fat meal induced endothelial damage in humans [65].

In our study, the activated MMP-2 gelatinolytic activity increased in MLP explants at 24 h in response to MPO treatment and was reduced with the treatment combination of MPO + ABAH. This may suggest that MMP-2 is implicated in an in vivo acute reaction to a MPO-induced inflammation in MLP endometria. In addition, MMP-9 active form gelatinolytic activity augmented with MPO treatment but was inhibited by ABAH at 48 h in FP endometrial explants. This could suggest MMP-9 participation, particularly, in FP, in a reaction to a continued exposure to MPO.

In our previous in vitro works, in response to elastase treatment, MMPs’ expression also differed according to the estrous cycle phase and treatment time, suggesting that the endometrial response is affected by hormonal variations and by the length of the stimulus [20]. Moreover, cathepsin G treatment increased MMPs gelatinolytic activity, mainly in follicular phase endometrial explants, and reduced with addition of a cathepsin G inhibitor [21]. The MMPs’ output differs and depends on the stimulus, phase of estrous cycle, and treatment duration. So, further in vitro and in vivo research is crucial to understand the action of MMPs in both healthy and fibrotic endometrium.

Therefore, future works should consider testing in vivo a combination of elastase, cathepsin G, and MPO inhibitors or the use of a single inhibitor capable of hindering all the pro-fibrotic effects of those enzymes found in NETs in mare endometrium. Based on this study, MMP-2 appears to be involved in a fast in vitro response to MPO treatment in MLP endometrial explants. In contrast, MMP-9 seems to be released by FP equine endometrial explants after a prolonged exposure to MPO.

## 5. Conclusions

Our findings reinforced the knowledge about MPO pro-fibrotic effects in equine endometrium. Myeloperoxidase induced COL1 and MMP-2/-9 activity in vitro in equine endometrium, and ABAH was shown to inhibit MPO-induced COL1 expression, as well as the activity of MMP-2/-9 induced by MPO. These data should be considered when studying endometrosis development and the attempt to fight this disease by inhibiting pro-fibrotic enzymes found in NETs. However, caution should be taken by not extrapolating these in vitro study results on the use of ABAH as an in vivo therapeutic approach to prevent endometrosis.

## Figures and Tables

**Table 1:** List of primers utilized in quantitative real-time polymerase chain reaction (qPCR).

Gene (Accession Number)	Sequence 5′-3′	Amplicon
*RPL32* (XM_001492042.6)	Forward: AGCCATCTACTCGGCGTCA	144
	Reverse: GTCAATGCCTCTGGGTTTCC	
*COL1A2* (XM_001492939.3)	Forward: CAAGGGCATTAGGGGACACA	196
	Reverse: ACCCACACTTCCATCGCTTC	
*MMP2* (XM_001493281.2)	Forward: TCCCACTTTGATGACGACGA	115
	Reverse: TTGCCGTTGAAGAGGAAAGG	
*MMP9* (NM_001111302.1)	Forward: GCGGTAAGGTGCTGCTGTTC	177
	Reverse: GAAGCGGTCCTGGGAGAAGT	

*RPL32*—ribosomal protein L32; *COL1A2*—collagen type 1 α2; *MMP2*—matrix metallopeptidase 2; *MMP9*—matrix metallopeptidase 9.

## Data Availability

No new data were created or analyzed in this study. Data sharing is not applicable to this article.

## Supplementary Materials

The following are available online at https://www.mdpi.com/2076-2615/11/1/208/s1: Figure S1: Activity of lactate dehydrogenase restrained in conditioned culture media of mare endometrial explants; Figure S2: Prostaglandin F2α secretion by mare endometrial explants after oxytocin treatment; Table S1: Significance levels (*p* values) for 2-, 3-, and 4-way interactions among estrous cycle phases, treatment time, and myeloperoxidase or 4-aminobenzoic hydrazide treatments for the measured variables; Table S2: Differences of the same treatments between the follicular phase and mid-luteal phase inside each treatment time; Figure S3: Representative panels of collagen type I western blotting and pro and active forms of MMP-2 and MMP-9 zymograms in mare endometrium.
