# Supplementary material for: Myeloperoxidase Inhibition Decreases the Expression of Collagen and Metallopeptidase in Mare Endometria under In Vitro Conditions
Source: Animals (Basel). 2021 Jan 16;11(1):208. doi: 10.3390/ani11010208 (PMC7830995; doi:10.3390/ani11010208)
Supplement: Supplementary file 1 [file animals-11-00208-s001.pdf]

Supplementary Figure S1

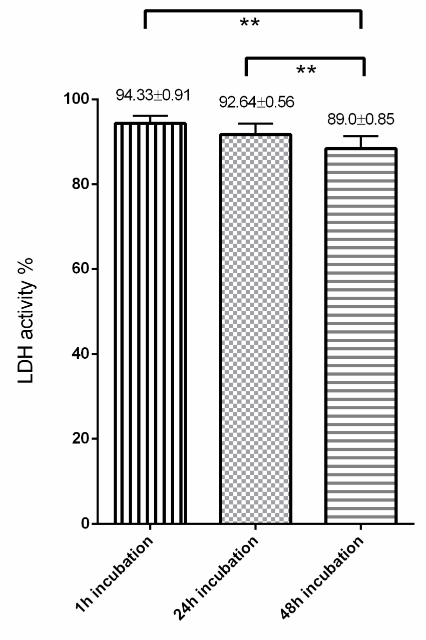

Supplementary Fig. S1: Lactate dehydrogenase (LDH) activity measured in conditioned culture medium of equine endometrial explants after 1h, 24h or 48h incubation. Explants viability was calculated from the quotient of the intracellular LDH activity and the total activity (extracellular plus intracellular LDH) [14]. Results are presented as means ± SEM. Asterisks indicate statistical differences within time of incubation. Statistical differences were found between 1h - 48 h, and 24h - 48h ($P < 0.01$). The results were independent of estrous cycle phase.

Supplementary Figure S2

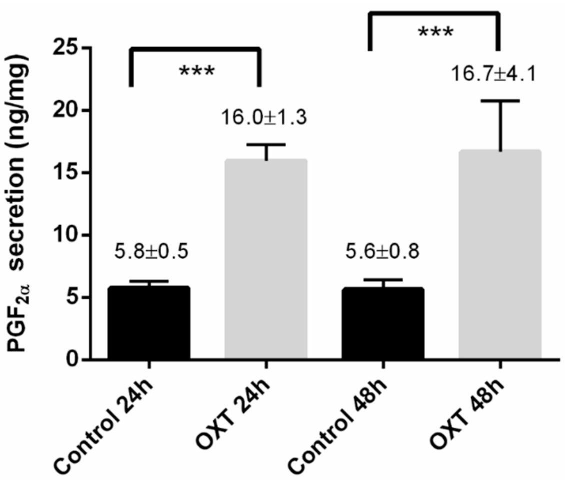

Supplementary Fig. S2: The effect of oxytocin (OXT) on prostaglandin (PG)$F_{2\alpha}$ secretion in equine endometrial explants after 24h or 48h. Results are presented as means ± SEM. Asterisks indicate statistical differences within the different time of treatment. Endometrial explants treatment with OXT increased $PGF_{2\alpha}$ secretion at 24h and 48h ($P > 0.001$) comparing to control (non-treated tissues). These results were independent of estrous cycle phase.

Supplementary Table S1

Supplementary Table S1: Levels of significance (P values) for 2-, 3- and 4-way interactions between estrous cycle phases, treatment time, and myeloperoxidase (MPO) or 4-aminobenzoic hydrazide (ABAH) treatments in the analyses of relative transcript of target genes, COL1 protein relative abundance and gelatinolytic activity of MMP-2 and -9. The results were considered significant at $P < 0.05$ and are highlighted in bold.

Interaction	*COL1A2*	COL1	*MMP2*	Pro-MMP-2	Active MMP-2	*MMP9*	Pro-MMP-9
MPO x ABAH	0.0982	0.1554	0.2915	0.216	**0.0187**	**0.0013**	0.5735
MPO x treatment time	0.9082	0.1717	0.6797	0.5799	0.7226	**0.0245**	0.4546
MPO x estrous cycle phase	**0.0292**	**0.0331**	0.4127	0.6731	0.6847	**0.0011**	0.5019
ABAH x treatment time	0.5321	0.3425	0.9631	0.2552	0.1017	0.3115	0.423
ABAH x estrous cycle phase	**0.0284**	0.0611	0.2415	0.3873	0.8572	**<.0001**	0.6101
Time of treatment x estrous cycle phase	**0.0006**	0.1814	0.6529	**0.007**	**0.044**	0.0572	**0.0131**
MPO x ABAH x treatment time	0.4935	0.497	0.8888	0.4357	0.9494	0.3593	0.0926
MPO x ABAH x estrous cycle phase	**0.0034**	**0.0245**	0.8832	0.153	0.1756	0.4073	0.8236
MPO x treatment time x estrous cycle phase	**0.0079**	0.491	0.7107	0.5299	0.2599	0.0875	0.8082
ABAH x treatment time x estrous cycle phase	0.8928	0.0689	0.655	0.1988	0.1769	0.3967	0.7737
MPO x ABAH x treatment time x estrous cycle phase	0.1748	0.8249	0.6073	**0.0065**	**0.0199**	**0.0856**	**0.0309**

Abbreviations: *COL1A2* - collagen type 1 α2; COL1 – collagen type I protein; *MMP2* - matrix metallopeptidase 2; *MMP9* - matrix metallopeptidase 9

Supplementary Table S2

Supplementary Table S2: Listed significant differences of the same treatments between the follicular phase (FP) and mid-luteal phase (MLP) of the estrous cycle, within each treatment time.

Evaluated variables	Treatment comparison	P value	Figures
COL1A2 transcription	MPO 24h FP *vs* 24h MLP	$P < 0.0001$	1A, 1B
COL1 protein relative abundance	MPO 24h FP *vs* 24h MLP	$P < 0.01$	2C, 2D
	MPO 48h FP *vs* 48h MLP	$P < 0.01$	
MMP9 transcription	ABAH 24h FP vs 24h MLP	$P < 0.001$	2C, 2D
	ABAH 48h FP vs 48h MLP	$P < 0.05$	
	MPO 24h FP *vs* 24h MLP	$P < 0.01$	
	MPO + ABAH 24h FP *vs* 24h MLP	$P < 0.0001$	
	MPO + ABAH 48h FP *vs* 48h MLP	$P < 0.0001$	
Pro-MMP-2 activity	MPO 24h FP *vs* 24h MLP	$P < 0.001$	3A, 3B
Active MMP-2	MPO 24h FP *vs* 24h MLP	$P < 0.01$	3A, 3B

COL1A2 - collagen type 1 α2; COL 1 – collagen type I; *MMP2* - matrix metallopeptidase 2; *MMP9* - matrix metallopeptidase 9; MPO – myeloperoxidase; ABAH – 4-aminobenzoic hydrazide; FP – follicular phase; MLP – mid-luteal phase.

Supplementary Fig. S3

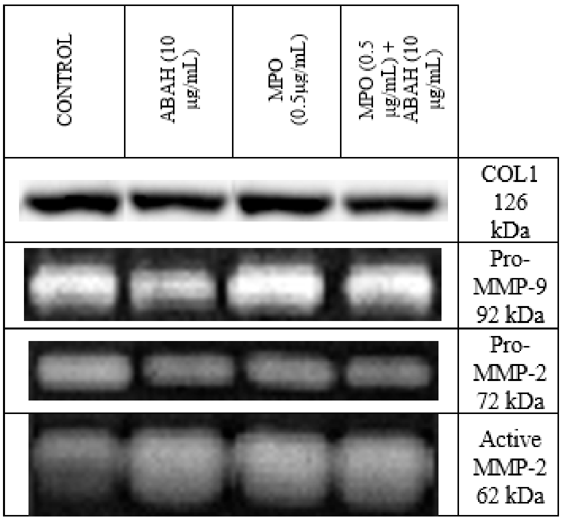

A – 24h FP endometrium explant

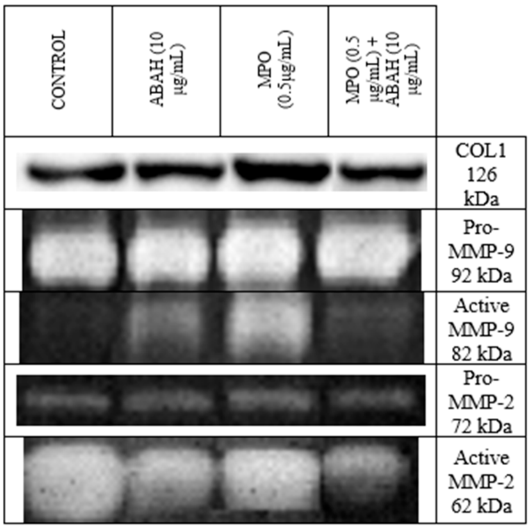

B – 48h FP endometrium explant

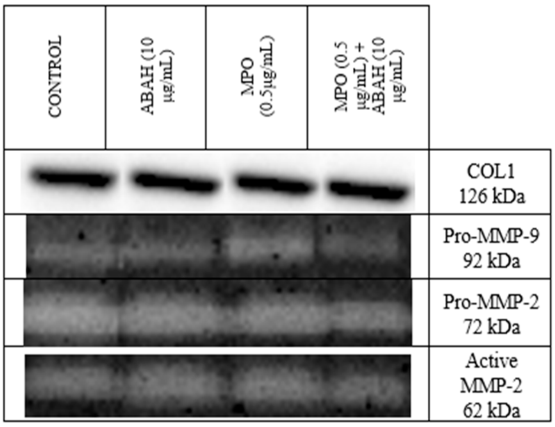

C – 24h MLP endometrium explant

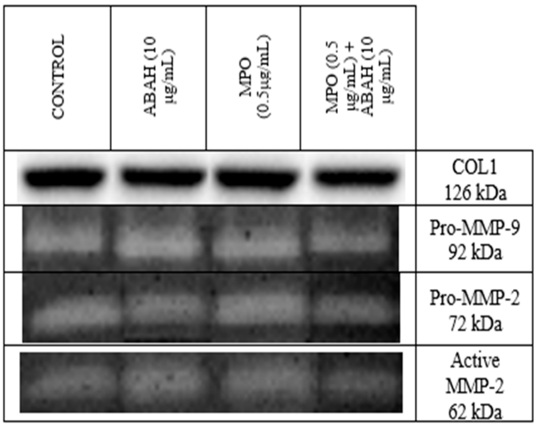

D – 48h MLP endometrium explant

Supplementary Fig. S3: Representative panels of type I collagen (COL1) western blotting and pro and active forms of MMP-2 and MMP-9 zymograms in mare endometrium in follicular phase (FP) or mid-luteal phase (MLP) treated for 24h or 48h with myeloperoxidase (MPO 0.5 µg/mL), 4-aminobenzoic hydrazide (ABAH; 10 µg/mL) or MPO (0.5µg/mL) + ABAH (10µg/mL). **A** - 24h treatment of FP endometrium explants; **B** – 48h treatment of FP endometrium explants, **C** – 24h treatment of MLP endometrium explants; and **D** – 48h treatment of MLP endometrium explants.
